# The Complexity and Entropy Analysis for Service Game Model Based on Different Expectations and Optimal Pricing

**DOI:** 10.3390/e20110858

**Published:** 2018-11-08

**Authors:** Yimin Huang, Xingli Chen, Qiuxiang Li, Xiaogang Ma

**Affiliations:** 1 The College of Management & Mconomics, North China University of Water Resources and Electric Power, Zhengzhou 450046, China; 2 Business School, Henan University, Kaifeng 475004, China; 3 The Institute of Management Science and Engineering, Henan University, Kaifeng 475004, China; 4 Management School, Wuhan Textile University, Wuhan 430073, China

**Keywords:** multichannel supply chain, service game, chaos, entropy, BOPS

## Abstract

The internet has provided a new means for manufacturers to reach consumers. On the background of the widespread multichannel sales in China, based on a literature review of the service game and multichannel supply chain, this paper builds a multichannel dynamic service game model where the retailer operates an offline channel and the manufacturer operates an online channel and offers customers the option to buy online and pick up from the retailer’s store (BOPS). The manufacturer and the retailer take maximizing the channel profits as their business objectives and make channel service game under optimal pricing. We carry on theoretical analysis of the model and perform numerical simulations from the perspective of entropy theory, game theory, and chaotic dynamics. The results show that the stability of the system will weaken with the increase in service elasticity coefficient and that it is unaffected by the feedback parameter adjustment of the retailer. The BOPS channel strengthens the cooperation between the manufacturer and the retailer and moderates the conflict between the online and the offline channels. The system will go into chaotic state and cause the system’s entropy to increase when the manufacturer adjusts his/her service decision quickly. In a chaotic state, the system is sensitive to initial conditions and service input is difficult to predict; the manufacturer and retailer need more additional information to make the system clear or use the method of feedback control to delay or eliminate the occurrence of chaos.

## 1. Introduction

In recent years, some Chinese retailers have profited enormously from the development of online marketing channels, however these profits have come at the expense of traditional retailers who once dominated the market. These changes in power of the channels have also created channel conflicts [1]. Scholars have studied pricing strategies in dual-channel supply chains from several perspectives. Huang et al. [2] considered the effects of production disruption and demand disruption on the pricing and production decisions in a dual-channel supply chain. Li et al. [3] studied the effects of green costs on the retail prices and green degrees of a competitive dual-channel green supply chain. Kouvelis et al. [4] discussed supply chain contracting in environments with volatile input prices and frictions, and presented a game-theoretic study of a bilateral monopoly supply chain with stochastic demand, stochastic input costs, production lead times, and working capital constraints. Li et al. [5] studied the effects of risk attitude of retailer and uncertain demand on the pricing strategy and coordination. Li et al. [6] studied the pricing strategy of a dual-channel supply chain considering a perishable product and risk preference. Radhi and Zhang [7] discussed the effects of customer preference and customer return rate on the pricing in a dual-channel supply chain. Li et al. [8] analyzed the effects of pricing strategy of the retailer on the manufacturer’s choice to open the direct selling channel. Ji et al. [9] developed four case models to study the optimal pricing and return policies considering false failure returns, and analyzed the influences of buy-back contract on optimal pricing and return policies. Xie et al. [10] built the revenue-sharing contract and cost-sharing contract to address the problem of forward channel conflicts and introduced the Stackelberg game to investigate the contract coordination mechanism. In contrast to this stream of work, this paper focuses on the service game of a multichannel supply chain where the manufacturer offers customers the option to buy online and pick up from the retailer’s store (BOPS), which is a new retail environment today.

Channel service is an important factor affecting customers’ channel choices and is broadly surveyed in the literature [11,12,13,14,15]. Considering service factors, Ma and Guo [16] studied the complex dynamics of a bivariate game model, in which the recursive least-squares (RLS) estimation is introduced to substitute naive estimation. Ali et al. [17] examined the effects of potential market demand disruptions on the prices and service levels of competitive retailers, and showed that the price and investment decision of service level are significant influenced by demand disruptions. Li and Li [18] found the entire supply chain could not be coordinated with a constant wholesale price when the retailer provided a value-added service and had concern for fairness. Zhou et al. [19] considered a dual-channel supply chain—where the retailer provided customers with certain pre-sales services and where the manufacturer free-rides the retailer’s pre-sales services by sharing the retailer’s sales effort cost—and investigated the influence of free riding on the pricing and service strategies of the two members. Zhou and Zhao [20] analyzed how the manufacturer used wholesale prices and slotting allowances to practice his signaling strategy with asymmetric information considering retailer’s value-added services, respectively. Chen et al. [21] studied a retail service supply chain with an online-to-offline (O2O) mixed channel under different power structures. Kong et al. [22] studied the pricing and service level of CLSC under centralized and decentralized decision-making, respectively, and analyzed the effects of system’s parameters on the system’s performance. The above papers studied the impacts of service on channel pricing strategy; no paper studies the channel service decision under optimal pricing.

There are channel conflicts between online and offline channels, such as inconsistent goals, business scopes, and consumer purchasing behavior. The new retail model enables for online embracing offline to achieve channel integration; many enterprises practice this new retail model according to their realities, such as Jing Dong, Tmall, and Uniqlo. Many scholars have also studied channel conflicts and cooperation contracts of O2O channel from different perspectives. Cai et al. [23] used the price discount contracts and pricing schemes to coordinate the dual-channel supply chain, and found price discount contracts and consistent pricing scheme can reduce channel conflict. Tao and Li [24] developed an O2O channel model and analyzed the influence of service level, the free-riding coefficient, and a bonus strategy on pricing policies and channel performance. Zhao et al. [25] investigated pricing problem of a dual-channel supply chain considering complementary products and different market power structures, and discussed the effects of important parameters on the pricing strategies.

Channel integration management has received a lot of attention in marketing; the topic was broadly surveyed in literature [26,27,28]. Jin et al. [29] studied BOPS theoretical model in which a physical retailer adopting BOPS used a recommended service area to fulfill orders from both online and offline customers; the size of the BOPS service area is determined by the ratio of unit inventory cost to BOPS customers. Liu and Zhou [30] found whether corporations adopt the BOPS model or not depended on the size of BOPS-consumer and consumer’s service sensitivity degree. Assuming a supply chain is comprised of a wholesaler and two retailers, Moon et al. [31] showed the process of collapsing the supply chain through interaction between subsystems by developing a system dynamics simulation model. Yan et al. [32] introduced the WeChat channel into multichannel supply chain system, and found that the WeChat channel could allow retailers to obtain increased profits and uncertainty for manufacturers. Matsui [33] investigated a multichannel supply chain model, where a manufacturer produces and sells products to retailers, and analyzed the optimal timing and level of wholesale and retail prices considering observable delay game.

The above literatures studied the impacts of service on channel conflicts and channel integration management. However, it is difficult for decision-makers to get all of the information in the market, so decision-makers have limited rational behavior. Because of their different expectations, few literatures have studied the service game of multichannel supply chain under the optimal price.

The supply chain system will be in an unstable state because of the manager’s behavior and customer’s behavior. Some scholars analyzed the complexity of supply chain based on entropy theory in literatures [34,35]. Kriheli and Levner [36] analyzed the complexity between the supply chain components under uncertainty environment using the information entropy. Levner and Ptuskin [37] presented the entropy-based optimization model for reducing the supply chain model size and assessing the economic loss. Lou et al. [38] analyzed the bullwhip effect in a supply chain with a sales game and consumer returns via the theory of entropy and complexity. Han et al. [39] built a duopoly game model with double delays in the hydropower market and analyzed the effect of time delay parameters on system entropy and stability.

The above researches are mainly focused on the pricing and service decisions of a dual-channel supply chain from the perspective of static operation. This paper will build a multichannel dynamic service game model and analyze its dynamic evolution characteristics using dynamics theory, game theory, and entropy theory.

The main contributions of this paper are as follows:

(1) This paper broadens and enriches the research of the multichannel service supply chain and proposes a new perspective for multichannel research and decision references for multichannel enterprises, because decision-makers hope to draw up service strategies for the multichannel supply chain to solve the practical troubles of firms;
(2) This paper studies the dynamic service strategy under optimal pricing which further widens the research scope of the multichannel supply chain;
(3) This paper uses the entropy theory and dynamics theory to study the complexity and characteristics of the multichannel service supply chain and reveals that decision variables and parameters have great impact on the stability of the multichannel service supply chain.

The rest of this paper is organized as follows. The model description and model construction are given in Section 2. Section 3 analyzes the stability of the system. Section 4 analyzes the complexity entropy and dynamic characteristics of the system. The feedback control model is designed to make the system return to the stable state in Section 5. Finally, Section 6 presents the conclusions of this paper.

## 2. Model Description and Model Construction

### 2.1. Model Description and Assumptions

**Figure 1:**
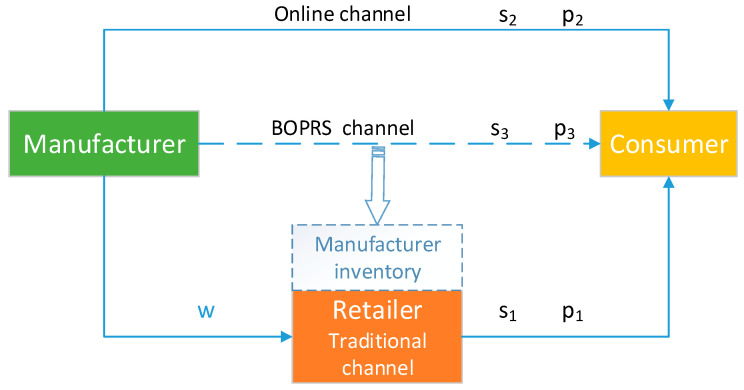
Multichannel service supply chain.

In this paper, a service game model is developed in which a retailer operates an offline channel and a manufacturer operates an online channel and offers customers the option to buy online and pick up at the retailer’s store (BOPS), as shown in Figure 1. The manufacturer and retailer take maximizing the channel profits as business objectives and make channel service game under optimal pricing, where $p_{i}$ and $s_{i},\;i=1,\;2,\;3$ represent the sale prices of the product and channel service levels in three channels and w is the wholesale price that the manufacturer offers to the retailer. The manufacturer and retailer allocate the cost and profit of BOPS channel in a certain proportion. The retailer does not participate in service decisions of BOPS channel. Therefore, it is reasonable for the manufacturer to make the service decision of BOPS channel based on channel profit.

The following assumptions are used to facilitate our model in this paper:

(1) The manufacturer and retailer aim at maximizing channel profits, the manufacturer takes $s_{2}$ and $s_{3}$ as decision variables and the retailer takes $s_{1}$ as a decision variable under the optimal price decision.
(2) Channel service does not affect the demands of other channels. The production costs and sales costs of products are zero.
(3) The inventories of the manufacturer and retailer are large enough to meet customer needs.

### 2.2. Model Construction

According to the actual market competition and extending the demand functions in Yao et al. [40] and Dan et al. [41], we assume that the primary demand functions in this paper are decided by $p_{i}$ and $s_{i}$ as follows

(1) $$\;\{\begin{array}{l} D_{1}=a_{1}-bp_{1}+kp_{2}+kp_{3}+\gamma_{1}s_{1}\\ D_{2}=a_{2}-bp_{2}+kp_{1}+kp_{3}+\gamma_{2}s_{2}\\ D_{3}=a_{3}-bp_{3}+kp_{2}+kp_{1}+\gamma_{3}s_{3} \end{array}\;$$

where $a_{i},\;i=1,\;2,\;3$ are the base demands of products for traditional channel, direct channel and BOPS channel. b (b>0) represents the price sensitive coefficient of the product; k (k>0) is the cross-price sensitivity coefficient which reflects the substitution degrees of the products; $\gamma_{i}\left(\gamma_{i}>0\right),\;i=1,\;2,\;3$ represent service sensitivity coefficients and satisfies $b>k\;\mathrm{and}\;b>\gamma_{i}$.

According to past literature [17], the service costs in the three channels satisfy: $c\left(s_{i}\right)=\frac{\eta_{i}s_{i}^{2}}{2},\;i=1,\;2,\;3$. $\eta_{i}>0\;\mathrm{and}\;i=1,\;2,\;3$ are unit service costs of each channel.

The decision-making process of the manufacturer and retailer is as follows (1) the manufacturer and retailer first make price decision simultaneously based on channel profits maximization and (2) then make service decision under the optimal price decision.

The channel profits of the manufacturer and retailer are represented as follows

(2) $$\;\{\begin{array}{l} \pi_{1}=\left(p_{1}-w\right)D_{1}-\frac{\eta_{1}s_{1}^{2}}{2}\\ \pi_{2}=p_{2}D_{2}-\frac{\eta_{2}s_{2}^{2}}{2}\\ \pi_{3}=p_{3}D_{3}-\frac{\eta_{3}s_{3}^{2}}{2} \end{array}\;$$

where $\pi_{1}$ is the retailer’s profit from the traditional channel and $\pi_{2}$ and $\pi_{3}$ are the manufacturer’ profits from online channel and BOPS channel. w is a constant which represents the wholesale price that the manufacturer provides for the retailer.

Supposing $s_{1}$, $s_{2}$, and $s_{3}$ are known, make a first-order partial derivative of $\pi_{i}$ for $p_{i}$, the channel marginal profits of the manufacturer and retailer are as follows

(3) $$\;\{\begin{array}{l} \frac{\partial \pi_{1}}{\partial p_{1}}=a_{1}-2bp_{1}+kp_{2}+kp_{3}+r_{1}s_{1}+bw\\ \frac{\partial \pi_{2}}{\partial p_{2}}=a_{2}-2bp_{2}+kp_{1}+kp_{3}+r_{2}s_{2}\\ \frac{\partial \pi_{3}}{\partial p_{3}}=a_{3}-2bp_{3}+kp_{1}+kp_{2}+\;r_{3}s_{3} \end{array}\;$$

By solving $\frac{\partial \pi_{i}}{\partial p_{i}}=0$, the optimal prices of the manufacturer and retailer are obtained:

(4) $$\left\{ \begin{array}{c} p_{1}^{*}=\frac{2a_{1}b-a_{1}k+a_{2}k+a_{3}k+2b^{2}w-bkw+2b\gamma_{1}s_{1}-k\gamma_{1}s_{1}+k\gamma_{2}s_{2}+k\gamma_{3}s_{3}}{2\left(b-k\right)\left(2b+k\right)}\\ p_{2}^{*}=\frac{2a_{2}b+a_{1}k-a_{2}k+a_{3}k+bkw+2b\gamma_{2}s_{2}+k\gamma_{1}s_{1}-k\gamma_{2}s_{2}+k\gamma_{3}s_{3}}{2\left(b-k\right)\left(2b+k\right)}\\ p_{3}^{*}=\frac{2a_{3}b+a_{1}k+a_{2}k-a_{3}k+bkw+2b\gamma_{3}s_{3}+k\gamma_{1}s_{1}+k\gamma_{2}s_{2}-k\gamma_{3}s_{3}}{2\left(b-k\right)\left(2b+k\right)}\; \end{array}\right.$$

Substituting $p_{1}^{*}$, $p_{2}^{*}$, and $p_{3}^{*}$ into the Equation (2), and making a first-order partial derivatives of $\pi_{i}$ for $s_{i}$. By solving the equations $\frac{\partial \pi_{i}}{\partial s_{i}}=0$, the optimal channel service levels of the manufacturer and the retailer are obtained as follows:

(5) $$\left\{ \begin{array}{c} s_{1}^{*}=\frac{A_{0}+\left(2b^{2}k\gamma_{1}\gamma_{2}-bk^{2}\gamma_{1}\gamma_{2}\right)s_{2}+\left(2b^{2}k\gamma_{1}\gamma_{3}-bk^{2}\gamma_{1}\gamma_{3}\right)s_{3}}{8\eta_{1}b^{4}—8k\eta_{1}b^{3}-4b^{3}\gamma_{1}^{2}-6\eta_{1}b^{2}k^{2}+4kb^{2}\gamma_{1}^{2}+4\eta_{1}bk^{3}-bk^{2}\gamma_{1}^{2}+2\eta_{1}k^{4}}\\ s_{2}^{*}=\frac{B_{0}+\left(2b^{2}k\gamma_{1}\gamma_{2}-bk^{2}\gamma_{1}\gamma_{2}\right)s_{1}+\left(2b^{2}k\gamma_{2}\gamma_{3}-bk^{2}\gamma_{2}\gamma_{3}\right)s_{3}}{8\eta_{1}b^{4}—8k\eta_{1}b^{3}-4b^{3}\gamma_{1}^{2}-6\eta_{1}b^{2}k^{2}+4kb^{2}\gamma_{1}^{2}+4\eta_{1}bk^{3}-bk^{2}\gamma_{1}^{2}+2\eta_{1}k^{4}}\\ s_{3}^{*}=\;\frac{C_{0}+\left(2b^{2}k\gamma_{1}\gamma_{3}-bk^{2}\gamma_{1}\gamma_{3}\right)s_{1}+\left(2b^{2}k\gamma_{2}\gamma_{3}-bk^{2}\gamma_{2}\gamma_{3}\right)s_{2}}{8\eta_{1}b^{4}—8k\eta_{1}b^{3}-4b^{3}\gamma_{1}^{2}-6\eta_{1}b^{2}k^{2}+4kb^{2}\gamma_{1}^{2}+4\eta_{1}bk^{3}-bk^{2}\gamma_{1}^{2}+2\eta_{1}k^{4}}\; \end{array}\right.$$

where

$$\begin{array}{c} \;A_{0}=4a_{1}\gamma_{1}b^{3}-4\gamma_{1}wb^{4}+a_{1}b\gamma_{1}k^{2}+2k\gamma_{1}b^{2}\left(a_{3}+a_{2}-2a_{1}\right)+\;b\gamma_{1}k^{2}\left(a_{3}-a_{2}\right)\\ +bk\gamma_{1}w\left(4b^{2}+3bk-2k^{2}\right) \end{array}$$

$$\begin{array}{c} \;B_{0}=4a_{2}\gamma_{2}b^{3}-a_{1}\gamma_{2}bk^{2}+2a_{1}k\gamma_{2}b^{2}+a_{2}b\gamma_{2}k^{2}-4a_{2}k\gamma_{2}b^{2}-a_{3}b\gamma_{2}k^{2}+2a_{3}k\gamma_{2}b^{2}\\ +2k\gamma_{2}wb^{3}-b^{2}k^{2}\gamma_{2}w\; \end{array}$$

$$\begin{array}{c} \;C_{0}=4a_{3}b^{3}\gamma_{3}-a_{1}bk^{2}\gamma_{3}+2a_{1}b^{2}k\gamma_{3}-a_{2}bk^{2}\gamma_{3}+2a_{2}b^{2}k\gamma_{3}+a_{3}bk^{2}\gamma_{3}-4a_{3}b^{2}k\gamma_{3}\\ +2b^{3}k\gamma_{3}w-b^{2}k^{2}\gamma_{3}w\; \end{array}$$

The expressions of the optimal service levels are very intricate; the relationship between variables and parameters cannot see intuitively from the expression functions. Next, we will structure a dynamic game model to research the dynamic characteristics of the multichannel supply chain system.

The service decisions of the manufacturer and the retailer are not completely rational because they cannot get all the necessary information in the market, so the manufacturer and the retailer have incomplete rational behavior when they make decisions. The retailer adopts an adaptive expectation in the decision-making process as follows

(6) $$\;s_{1}\left(t+1\right)=s_{1}\left(t\right)+\beta \left[s_{1}\left(t\right)-s_{1}^{*}\left(t\right)\right],\;\;\;\;\;\;\;0<\beta <1\;$$

where β is the service feedback parameter.

The manufacturer makes service decision based on bounded rationality expectation for direct channel and static expectation for BOPS channel:

(7) $$\;s_{2}\left(t+1\right)=s_{2}\left(t\right)+\xi s_{2}\left(t\right)\frac{\partial \pi_{2}\left(t\right)}{\partial s_{2}}\;$$

(8) $$\;s_{3}\left(t+1\right)=s_{3}^{*}\left(t\right)\;$$

where ξ is the service adjustment parameter which reflects the manufacturer’s learning behavior and positive managerial behavior. When the marginal profit in period t exceeds zero, the manufacturer will increase the service level in period t+1; contrarily, the manufacturer will decrease the service level in period t+1. Namely, the service level of period t+1 will be adjusted according to marginal profit of period t.

The three-dimensional dynamic service game system considering BOPS channel is as follows

(9) $$\left\{ \begin{array}{c} s_{1}\left(t+1\right)=s_{1}\left(t\right)+\beta \left[\frac{A_{0}+\left(2b^{2}k\gamma_{1}\gamma_{2}-bk^{2}\gamma_{1}\gamma_{2}\right)s_{2}\left(t\right)+\left(2b^{2}k\gamma_{1}\gamma_{3}-bk^{2}\gamma_{1}\gamma_{3}\right)s_{3}\left(t\right)}{8\eta_{1}b^{4}-8\eta_{1}b^{3}k-4b^{3}\gamma_{1}^{2}-6\eta_{1}b^{2}k^{2}+4b^{2}k\gamma_{1}^{2}+4\eta_{1}bk^{3}-bk^{2}\gamma_{1}^{2}+2\eta_{1}k^{4}}-s_{1}\left(t\right)\right]\\ s_{2}\left(t+1\right)=s_{2}\left(t\right)+\xi s_{2}\left(t\right)\left[\frac{B_{1}+B_{2}s_{1}\left(t\right)+B_{3}s_{2}\left(t\right)+\left(4b^{2}k\gamma_{2}\gamma_{3}-2bk^{2}\gamma_{2}\gamma_{3}\right)s_{3}\left(t\right)}{{\left(2b+k\right)}^{2}{\left(2b-2k\right)}^{2}}-\eta_{2}s_{2}\left(t\right)\right]\\ s_{3}\left(t+1\right)=\frac{C_{0}+\left(2b^{2}k\gamma_{1}\gamma_{3}-bk^{2}\gamma_{1}\gamma_{3}\right)s_{1}\left(t\right)+\left(2b^{2}k\gamma_{2}\gamma_{3}-bk^{2}\gamma_{2}\gamma_{3}\right)s_{2}\left(t\right)}{8\eta_{3}b^{4}-8\eta_{3}b^{3}k-4b^{3}\gamma_{3}^{2}-6\eta_{3}b^{2}k^{2}+4b^{2}k\gamma_{3}^{2}+4\eta_{3}bk^{3}-bk^{2}\gamma_{3}^{2}+2\eta_{3}k^{4}}\;\; \end{array}\right.$$

where

$B_{1}=8a_{2}\gamma_{2}b^{2}\left(b-k\right)+2bk\gamma_{2}(a_{1}k-wbk+2wb^{2}+4k\gamma_{2}b^{2}\left(a_{1}+a_{3}\right)-2b\gamma_{2}k^{2}\left(a_{1}+a_{3}\right),$
$B_{2}=2bk\gamma_{1}\gamma_{2}\left(2b-k\right),$
$B_{3}=2bk^{2}\gamma_{2}^{2}+8b^{2}\gamma_{2}^{2}\left(b-k\right).$

## 3. The Stability of System (9)

In this section, we will study the stable characteristics of system (9). Because of the particularity of the model, the Nash equilibrium solutions of system (9) are very complicated, and we cannot judge directly the interaction between variables and parameters. Here, we will study the stability of system (9) through numerical simulation [42], according to the current state and reality of the multichannel supply chain enterprises, the parameter values are as follows, $a_{1}=4$, $a_{2}=3$, $a_{3}=2$, b=1, $\eta_{1}=0.7$, $\eta_{2}=0.75$, $\eta_{3}=0.7$, $\gamma_{1}=0.55$, $\gamma_{2}=0.4$, $\gamma_{3}=0.5$, k=0.6, w=2.

When $s_{1}\left(t+1\right)=s_{1}\left(t\right)$, $s_{2}\left(t+1\right)=s_{2}\left(t\right)$, $s_{3}\left(t+1\right)=s_{3}\left(t\right)$, the eight equilibrium solutions of system (9) are,

$E_{1}=\left(9.5227,\;6.3236,\;8.7891\right),$
$E_{2}=\left(0,\;4.6685,\;6.3699\right),$
$E_{3}=\left(7.9027,\;0,\;7.3865\right),$
$E_{4}=\left(7.0220,\;4.8424,\;0\right),$
$E_{5}=\left(6.0508,\;0,\;0\right),$
$E_{6}=\left(0,\;3.859,\;0\right),$
$E_{7}=\left(0,\;3.8509,\;0\right),$
$E_{8}=\left(0,\;0,\;5.6042\right).$

Obviously, $E_{2}$, $E_{3}$, $E_{4}$, $E_{5}$, $E_{6}$, $E_{7}$, and $E_{8}$ are boundary equilibrium points which do not meet our expectations, because the decision variables obviously are not allowed to be zero in economics for decision makers, $E_{2}–E_{8}$ are unstable and $E_{1}$ is the only Nash equilibrium point. Because it is not significant to study the unstable equilibrium points we only consider the stability of the Nash equilibrium point in the following.

The Jacobian matrix of system (9) at $E_{1}$ is as follows

$$\;J\left(E_{1}\right)=\left|\begin{array}{ccc} 1-\beta & 0.2621\beta & 0.3277\beta \\ 0.9611\xi & 1-10.4062\xi & 0.8737\xi \\ 3.3863 & 1.8452 & 0 \end{array}\right|\;$$

The characteristic polynomial of $J\left(E_{1}\right)$ takes the following form:

(10) $$\;f\left(\lambda \right)=\lambda^{3}+A\lambda^{2}+B\lambda +C\;$$

where

A=β+3.820ξ−2,
B=3.6164βξ−1.0560β−3.9052ξ+1,
C=−0.3410βξ+0.0565β+0.0805ξ.

In order to guarantee $E_{1}$ is locally stable, A, B, and C must meet the following conditions.

(11) $$\;\{\begin{array}{l} f\left(1\right)=A+B+C+1>0\;\;\\ \begin{array}{l} -f\left(-1\right)=-A+B-C+1>0\\ \left|C\right|<1\; \end{array}\\ \left|BC-AB\right|<\left|C^{2}-A^{2}\right| \end{array}\;$$

By solving condition (11), the stability domain of system (9) can be obtained. Due to these limitations being so complex, solving the inequality of Equation (11) is very difficult. If $E_{1}$ satisfies the inequality of Equation (11), we may judge that system (9) is locally stable. We will prove the stable region of system (9) through numerical simulation.

**Figure 2:**
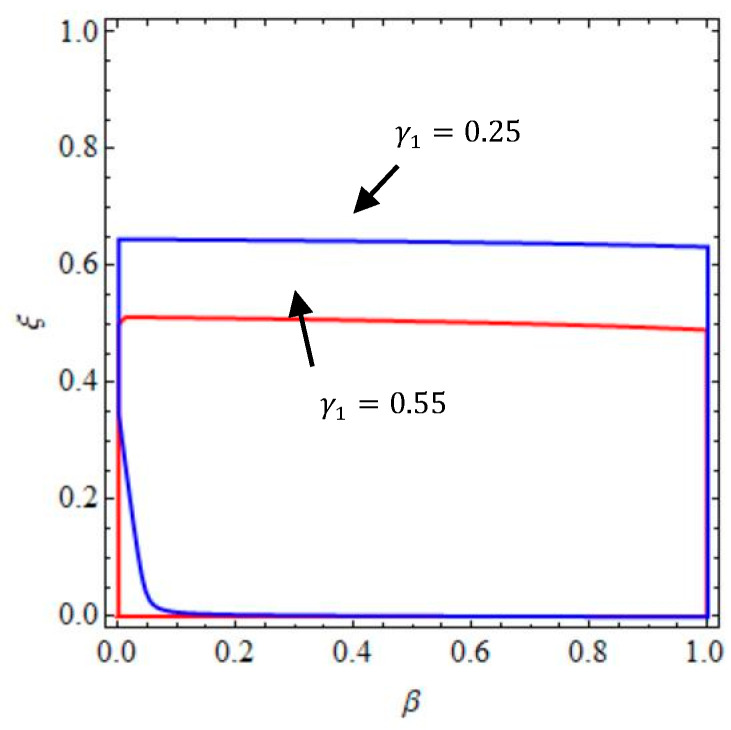
The stable regions of system (9) with $\gamma_{1}=0.25$ and $\gamma_{1}=0.55$.

According to the inequality Equation (11), Figure 2 gives the stable region and unstable region of system (9) when $\gamma_{1}=0.25$ and $\gamma_{1}=0.55$.

We can see that the stable region of system (9) becomes smaller with increasing $\gamma_{1}$, the change of β has no effect on the stability of system (9) no matter what value the $\gamma_{1}$ takes. Namely, the greater the service elasticity coefficient is, the smaller the stable region is; the increase of the service elasticity coefficient will weaken the market competition; thus, the choice of irrational decision-making mode has a great influence on the stability of system (9). When ξ and β take values in the stable range, the system (9) will stabilize at Nash equilibrium point after a finite period game. When ξ and β take values in the unstable range, the system (9) will be unstable and enter into either a bifurcation state or chaotic state, the uncertainty of system (9) increases at this time and more information is needed to maintain the stability of the system (9).

## 4. The Entropy Complexity Analysis of System (9)

In order to better understand the dynamic characteristics of system (9), in this section, numerical simulation is used to explore the entropy complexity and dynamic behavior of system (9) using the bifurcation diagram, system’s entropy, and the largest Lyapunov exponents (LLE), etc.

We know that entropy can measure the chaotic degree of the system; the system entropy is small when the system is in stable state and the system entropy is large when the system is in chaotic state. The equation of entropy used in this paper is as follows

$$\;S\left(p_{1},\;p_{2},\;\cdots ,p_{n}\right)=-\overset{n}{\underset{i=1}{\sum }}p_{i}\log_{2}p_{i}\;$$

Inevitably there will be many uncertain factors in the complex and changeable market. In this paper, through simulation analysis, we can clearly see the effect of parameter changing on the entropy of the system of the dual-channel supply chain, and then quantify the stability of the supply chain system using entropy, which lays the foundation for further effectively controlling the complexity of the whole supply chain.

### 4.1. The Entropy Complexity Analysis of System (9) with the Change of ξ

**Figure 3:**
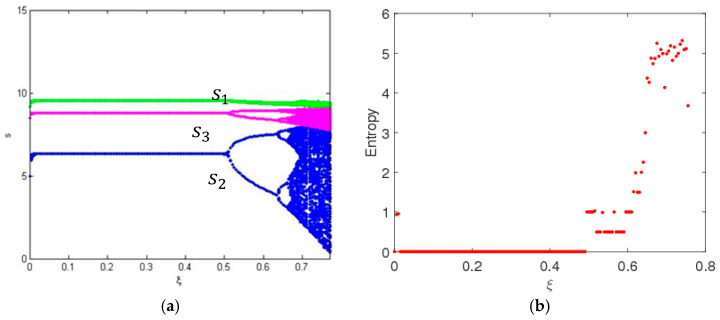
The evolution process of system (9) with change of ξ when $\gamma_{1}=0.55$. (**a**) The bifurcation diagram and (**b**) the entropy diagram.

In this part, we also suppose the parameters values as above, the entropy and dynamic behavior of system (9) are described with the change of ξ when β = 0.3. Figure 3 shows the service level and the entropy of system (9) as ξ changes with $\gamma_{1}=0.55$. We can see that, for the multichannel supply chain system in this paper, the service level in traditional selling channel is the highest and the one of direct channel is the lowest, which accords with the operation of the real market. System (9) is stable when ξ<0.49, and the bifurcation and chaos in system (9) occur through period-doubling bifurcation when ξ increases. System (9) has low entropy when it is in a stable state, and has high entropy when it is in a chaotic state. High entropy implies the system is more unstable; there exist many uncertainties in the complex and changeable market, and we require more information to keep system (9) in a stable state.

**Figure 4:**
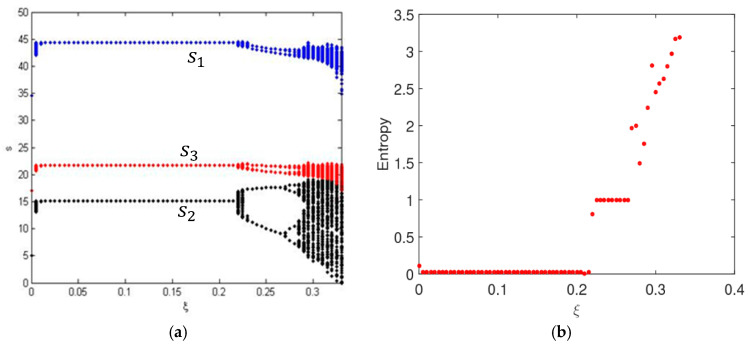
The evolution process of system (9) with change of ξ with $\gamma_{1}=0.7$. (**a**) The bifurcation diagram and (**b**) the entropy diagram.

Figure 4 shows the service levels and the entropy of system (5) as ξ changes with $\gamma_{1}=0.7$. We find that when consumers are more sensitive to service levels, system (9) will stabilize at a higher Nash equilibrium point, and the uncertainty of system (9) will appear earlier with high entropy, which is agreement with Figure 2. In the chaotic state, market competition is complex and unpredictable.

In short, when consumers are more sensitive to channel services, the manufacturer and retailer will provide higher levels of channel services, and the uncertainty of system (9) will appear earlier with high entropy. In other words, the greater the service elasticity coefficient is, the easier system (9) goes into a chaotic state and the smaller the system’s stable region becomes.

### 4.2. The Entropy Complexity Analysis of System (9) with Feedback Parameter (β)

**Figure 5:**
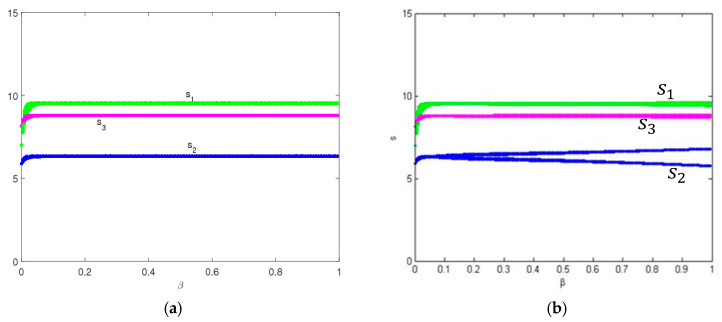
The bifurcation diagrams of the system (9) with change of β. (**a**) ξ=0.3 and (**b**) ξ=0.5.

In this section, we analyze the effects of feedback parameter (β) on the stability of system (9). Figure 5 is the bifurcation diagrams of system (9) with β changing. From Figure 5a, when ξ=0.3, the system (9) gradually returns to the Nash equilibrium from the initial value no matter how β changes, system’s entropy is zero and the market is in a stable state at this time. From Figure 5b, the system (9) is in the stable state with 0<β≤0.08 and makes 2-period bifurcation with 0.08<β≤1. So it can be seen that the feedback parameter (β) has little effect on system stability and system entropy.

**Figure 6:**
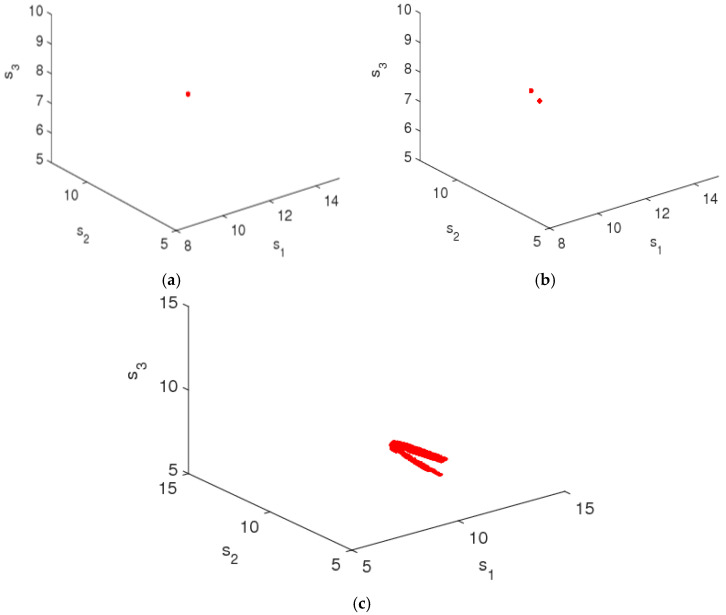
Chaotic attractor of system (9), (**a**) β=0.3, ξ=0.48; (**b**)  β=0.3, ξ=0.58; and (**c**)  β=0.3, ξ=0.74.

When the system is in a stable state, the system’s attractor is stable at fixed point; when the system goes into a chaotic state, the system’s attractor will occupy a larger space and the structure of the chaotic attractor will be more complicated. When β=0.3 and ξ=0.48, system (9) is in stable state according to the bifurcation diagram shown in Figure 3, the chaotic attractor in this condition is shown in Figure 6a; when β=0.3 and ξ=0.58, the system (9) is in 2-period bifurcation state, the chaotic attractor is shown in Figure 6b. When β=0.3 and ξ=0.74, the system (9) is in the chaotic state and the chaotic attractor is shown in Figure 6c.

From Figure 2, Figure 3, Figure 4, Figure 5 and Figure 6, it can be seen that for the multichannel supply chain, the change of ξ can affect the period of the market entering chaos state even if the initial value of service variable is fixed. The more quickly the manufacturer adjusts channel service, the more easily the market falls into chaos. Therefore, in this competitive multichannel supply chain, the manufacturer and retailer should make their decisions with an overall consideration about the market situation and competitor’s response rather than adjust their service level quickly and blindly.

According to information theory, when the market is in an orderly competitive state, the probability of optimal service level under optimal pricing will be large and system entropy will be low; when the market is in a disorderly competitive state, the service decisions under optimal pricing are out of order and the system entropy will be high.

**Figure 7:**
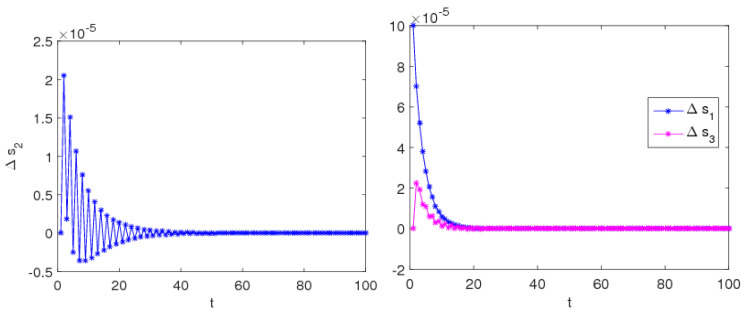
Initial value sensitivity of service level in stable system.

Another obvious feature of the chaotic system is sensitive to the initial values. In other words, if there is a slight change in the initial values of the system’s parameters, the results of system evolution will change greatly with time. When β=0.3 and ξ=0.48, system (9) stays stable according to the above analysis. The initial values of the service level ($s_{2}$) are taken 5 and 5.001, after multiple iterations the differences between the two sets of numerical solutions are shown in Figure 7. We can find that, at the beginning of iterations, there is a little difference, but after approximately 40 iterations, the difference gradually reduces to zero.

**Figure 8:**
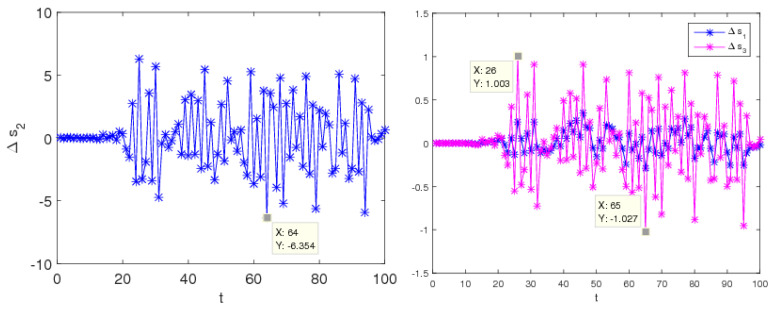
Initial value sensitivity of service level in a chaotic system.

When β=0.3 and ξ=0.72, system (9) stays in a chaotic state according to the above analysis. The initial values of the service level ($s_{2}$) are taken 5 and 5.001, after multiple iterations, the differences between the two sets of numerical solutions are shown in Figure 8. We can see that, during the initial iterations, the values of channel services are no difference, but after approximately 20 iterations, the differences in channel services increase greatly.

Thus, it can see that the system is very sensitive to the initial value when the system is in chaos; small differences in initial values can cause a huge deviation after multiple iterations, which give us reassurance that decision-makers should choose the initial values of their decision variables more prudently.

### 4.3. The Influence of Parameter Changes on System’s Profit

**Figure 9:**
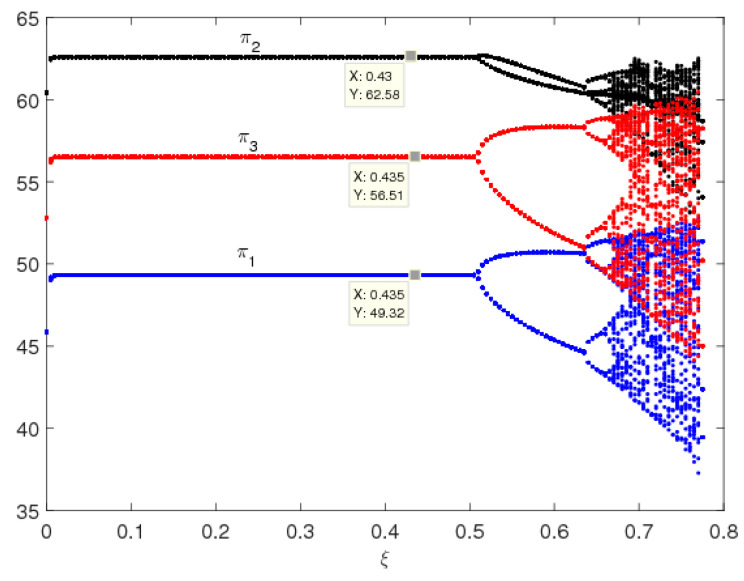
Profit diagram of system (9).

**Figure 10:**
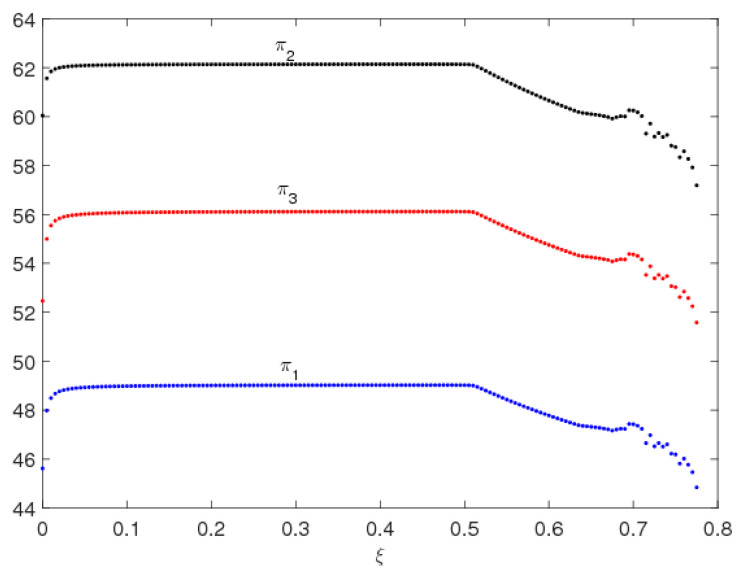
Average profit diagram of system (9).

From the analysis above, we can find that when ξ is oversized, the uncertainty of system (9) will increase obviously, which can cause the market be complex and increase the system’s entropy to a very large value making it difficult for decision-makers to make service decisions. So, we suspect that the profits of the system’s participators will also be influenced. Figure 9 and Figure 10 show the profit bifurcation diagrams of system (9) with respect to the change of ξ, respectively. As we have predicted, the profits of the system (9) stay stable when ξ<0.48, and enter into a chaotic state when ξ>0.68; in the chaotic state, the average profits of the system (9) show a downward trend (see in Figure 10). Therefore, the oversize of ξ will make the decision making complicated and affect the profits of the manufacturer and retailer.

Moreover, the retailer in traditional channel makes the largest service level, but gets the smallest profit in channel competition, which causes a conflict between online and offline channels. However, the manufacturer and retailer build a profit distribution contract of BOPS channel, which moderates the conflict between the online and the offline channels.

## 5. Chaos Control

In a multichannel supply chain, all administrators want to achieve their own business goals easily and adjust their service strategies frequently to reduce uncertainty in market competition. Once the adjustment speeds of service levels carry out in an irrational state, the market will be out of order and fall into chaos, which is harmful to the manufacturer and retailer. The manufacturer and retailer need more information to eliminate the uncertainty of the system. Consequently, some methods should adopt to defer or remove the occurrence of bifurcation and chaos.

The above analysis shows that the change of ξ makes the multichannel supply chain system enter chaotic state gradually. In this section, the feedback control method is be used to delay or eliminate the chaotic behavior of the multichannel service game model, thus reduce the negative impact of chaos on the system (9). Wu and Ma [43] have used the feedback control method to control chaos in the product horizontal diversification supply chain.

The original dynamic system (9) is as follows

(12) $$\;\{\begin{array}{c} s_{1}\left(t+1\right)=f_{1}\left[s_{1}\left(t\right),s_{2}\left(t\right),s_{3}\left(t\right)\right]\\ s_{2}\left(t+1\right)=f_{2}\left[s_{1}\left(t\right),s_{2}\left(t\right),s_{3}\left(t\right)\right]\\ s_{3}\left(t+1\right)=f_{3}\left[s_{1}\left(t\right),s_{2}\left(t\right),s_{3}\left(t\right)\right] \end{array}\;$$

The controlled dynamic system can be expressed as follows

(13) $$\;\{\begin{array}{l} s_{1}\left(t+1\right)=f_{1}\left[s_{1}\left(t\right),s_{2}\left(t\right),s_{3}\left(t\right)\right]\\ s_{2}\left(t+1\right)=f_{2}\left[s_{1}\left(t\right),s_{2}\left(t\right),s_{3}\left(t\right)\right]-Ps_{2}\left(t\right)\\ s_{3}\left(t+1\right)=f_{3}\left[s_{1}\left(t\right),s_{2}\left(t\right),s_{3}\left(t\right)\right] \end{array}\;$$

where P represents the chaos control parameter. Selecting an appropriate value for P is essential to delay bifurcation, which can make the multichannel supply chain system return to the stable state from the chaotic state.

**Figure 11:**
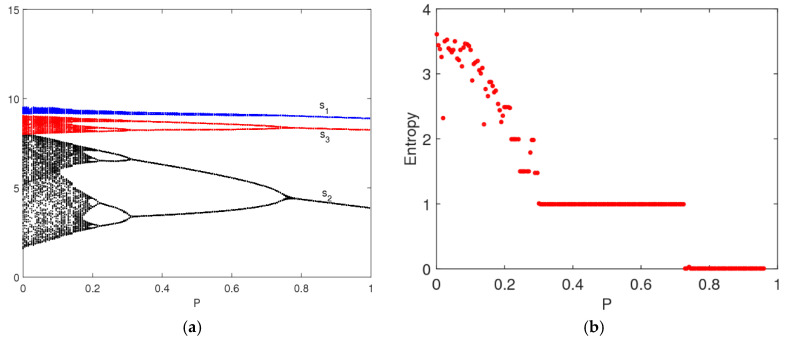
The evolution process of controlled system with P changing. (**a**) The bifurcation diagram and (**b**) the entropy diagram.

Figure 11 shows the bifurcation diagram and entropy diagram of the controlled system (13) with the change of P when β=0.3 and ξ=0.72. The controlled system (13) gradually enters the stable state from the chaotic state with the increase of P; when 0.73≤P≤1, the controlled system (13) is in the stable state and has low entropy in which the market is in an orderly competitive state.

In the real market, the control parameter P can act as an external interference for decision-makers for the multichannel supply chain system. When the multichannel supply chain goes into a chaotic state with the increase in market uncertainty, the decision maker should actively intervene in market competition. In other words, the control parameter P can also regard as a decision-maker’s learning and self-adaptive ability. The decision-makers can select appropriate values for P to achieve the multichannel supply chain back to the stable state.

## 6. Conclusions

In this paper, we build a multichannel dynamic service game model where a retailer operates an offline channel and a manufacturer operates an online channel and offers customers the option to buy online and pick up in retailer’s store (BOPS). The manufacturer and retailer maximize the channel profits as a business objective and make the channel service game under optimal pricing. The equilibrium solutions, the stable region, complexity entropy, and efficiency of the multichannel supply chain system are studied. The results show that the stability of the multichannel supply chain system will weaken with the service elasticity coefficient increasing and almost be unaffected by the feedback parameter adjustment of the retailer. If the manufacturer adjusts his service decision quickly, the system will go into a chaotic state, the average profits of the system will show a downward trend, and the system’s entropy will increase. The BOPS channel strengthens the cooperation between the manufacturer and retailer and moderates the conflict between the online and the offline channels. In chaos, the service input is difficult to predict and the system entropy is large, the manufacturer and retailer need more information to familiarize themselves with the market environment. The chaotic system can be delayed or eliminated effectively using the method of feedback control, so that the manufacturer and retailer should cooperate with each other to make some measures to prevent and control chaos.

The conclusions of this paper involve the elucidation of a realistic guide for the manufacturer and retailer to make optimal service decisions to avoid chaos and profit loss. For example, the manufacturer should have sufficient information to analyze consumers’ sensitivity to channel services. If the manufacturer blindly adjusts the channel service levels, it is easy to increase the uncertainty of the system and make the market go into chaos. In chaos, the manufacturer and retailer suffer unnecessary losses, and may even exit the market.

Nonetheless, some assumptions made in this paper limit the research results of the model. Loosing these assumptions can make the model close to market operation scenarios more. For instance, a model considered the interaction effect of service on channel demand will close to the actual situation. Second, other power structures between the manufacturer and retailer in the multichannel supply chain should take into account, as it may shed light on whether the current results will hold. Finally, the other decision objectives of the manufacturer and retailer should consider the future. We hope that the ideas and the model presented in this paper will lay the motivational ground for future research in these directions.
